# Physics of Protein Aggregation in Normal and Accelerated Brain Aging

**DOI:** 10.1002/bies.70030

**Published:** 2025-06-20

**Authors:** Alberto J. Espay, Andrea Sturchio, Alberto Imarisio, Emily J. Hill, Brady Williamson, Kora Montemagno, Christian Hoffmann, Hugo Le Roy, Dragomir Milovanovic, Fredric P. Manfredsson

**Affiliations:** ^1^ James J. and Joan A. Gardner Family Center for Parkinson's Disease and Movement Disorders Department of Neurology University of Cincinnati Cincinnati Ohio USA; ^2^ Section of Neurology Karolinska Institutet Stockholm Sweden; ^3^ Department of Molecular Medicine University of Pavia Pavia Italy; ^4^ Neurogenetics Research Centre, IRCCS Mondino Foundation Pavia Italy; ^5^ Department of Radiology University of Cincinnati College of Medicine Cincinnati Ohio USA; ^6^ Center for functionally integrative neuroscience Institute for Clinical Medicine Aarhus University Aarhus Denmark; ^7^ Laboratory of Molecular Neuroscience German Center for Neurodegenerative Diseases (DZNE) Berlin Germany; ^8^ Institute of Physics, School of Basic Sciences École Polytechnique Fédérale de Lausanne Lausanne Switzerland; ^9^ Einstein Center for Neuroscience Charité‐Universitätsmedizin Berlin, Corporate Member of Freie Universität Berlin and Humboldt‐Universität Berlin Berlin Germany; ^10^ Department of Translational Neuroscience and the Muhammad Ali Parkinson Center Barrow Neurological Institute Phoenix Arizona USA

**Keywords:** amyloid, Alzheimer's disease, cross‐beta, nucleation, Parkinson's disease, seed amplification assay, supersaturation

## Abstract

Protein aggregation is a normal response to age‐related exposures. According to the thermodynamic hypothesis of protein folding, soluble proteins precipitate into amyloids (pathology) under supersaturated conditions through a process similar to crystallization. This soluble‐to‐insoluble phase transition occurs via nucleation and may be catalyzed by ectopic surfaces such as lipid nanoparticles, microbes, or chemical pollutants. The increasing prevalence of these exposures with age correlates with the rising incidence of pathology over the lifespan. However, the formation of amyloid fibrils does not inherently cause neurodegeneration. Neurodegeneration emerges when the levels of functional monomeric proteins, from which amyloids form, fall below a critical threshold. The preservation of monomeric proteins may explain neurological resilience, regardless of the extent of amyloid deposition. This biophysical framework challenges the traditional clinicopathological view that considers amyloids intrinsically toxic, despite the absence of a known mechanism of toxicity. Instead, it suggests that chronic exposures driving persistent nucleation consume monomeric proteins as they aggregate. In normal aging, replacement matches loss; in accelerated aging, it does not. A biophysical approach to neurodegenerative diseases has important therapeutic implications, refocusing treatment strategies from removing pathology to restoring monomeric protein homeostasis above the threshold needed to sustain normal brain function.

## Graphical abstract

Soluble monomeric proteins precipitate via nucleation into insoluble amyloids in response to age‐related exposures (e.g., microbes, nanoparticles). Persistent soluble‐to‐insoluble phase transition depletes the functional proteins. In normal aging, replacement matches loss; in accelerated aging, it does not. Restoring monomeric protein homeostasis—not clearing amyloids—may slow progression in neurodegenerative diseases (figure was created using Biorender).

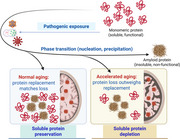

## 1 Introduction

Neurodegeneration research has been inspired by a clinicopathological model whereby pathology begets neurodegeneration and explains clinical features. Brain tissue at autopsy has not only rendered the final neurological diagnosis for anyone with symptoms of accelerated neurological aging but has long provided a forensic narrative in which whatever appeared abnormal under a microscope must be the source of the problem, not its consequence. The microscopic clumps represented by amyloids, proteins in an aggregated cross‐β pleated state, came to be referred to as pathology. In short order, *pathology* meant *pathogenesis*, and neurodegenerative disorders became *proteinopathies, *disorders caused by the toxic effects of proteins.

Physical principles applied to protein folding and aggregation provide an alternative framework to the research on brain aging. The last two decades have been dominated by the pursuit of understanding how pathology “spreads” from one brain region to another. Our medical lexicon prominently features such descriptors as “self‐propagating pathogenic protein aggregates” [1], “neuron‐to‐neuron spread of toxic fibers” [2], and “prion‐like replication” [3] with the adjective “toxic” commonly attached to proteins in a non‐native state (e.g., “toxic amyloid,” “toxic oligomers”). Removing aggregated proteins or preventing their assumed pathogenic spread has been widely conceived as a critical part of the solution to neurodegenerative disorders.

In this review, we discuss an alternative to the prion‐like explanation for pathology spread (whereby misfolded proteins propagate in an uncontrolled manner), making the case that the soluble‐to‐insoluble phase transitions of proteins, which ends in what we recognize as pathology, can be explained by the physics of crystal nucleation and growth, which follows thermodynamic equilibrium. We propose replacing the descriptive clinicopathologic framework that has been used to explain neurodegeneration for the last century with a biophysical framework, according to which pathology is not inherently toxic but the result of a nucleation event, catalyzed by the conditions of the microenvironment, not by a particular amino acid sequence. Lastly, we propose that the pathophysiology of accelerated brain aging is dependent on the loss of the normal, monomeric proteins as they precipitate into pathology, not the accrual of such pathology or the two‐dimensional cross‐β shape of its fibers.

## 2 Definitions

Protein aggregation involves a structural shift in protein homeostasis—from a soluble to an insoluble state. This transition typically occurs from a native state (such as monomeric or “intrinsically disordered” proteins, which exhibits various structures depending on their interactions) to an aggregated state, often representing a diverse range of conformations, among which the amyloid state is the most well‐known and the easiest to detect experimentally [4, 5]. While amyloid structures can vary infinitely in their two‐dimensional arrangements, they share a common cross‐β sheet conformation (see Glossary in Box 1).

**BOX 1. Glossary**

**Cross‐β conformation**: Two β sheets that are comprised of repeating protein monomer units separated by a 4.8 Å distance, which interact with each other via interdigitating sidechains (steric zipper).

**Amyloids**: Protein solids with cross‐β conformation.

**Protofilament**: Extended β‐sheet pairs that form an independent unit.

**Amyloid fibril**: One or more protofilaments associated laterally.

**Supersaturation**: the state in which a protein is dissolved in a solution at a concentration beyond its maximum (equilibrium) solubility.

**Nucleation**: The formation of the first stable amyloid fragment within a supersaturated protein solution.

**Polymorphs**: Different cross‐sectional shapes of protofilaments or fibrils.

Much of the scientific literature focuses heavily on the amyloid end of this process—often referred to as *pathology* in clinical contexts—due to the wide availability of measurement tools specifically suited for amyloids, such as x‐ray diffraction, or chemical dye. This methodological preference introduces an observational bias toward amyloids, suggesting a causal role of amyloid fibers in neurodegeneration [6, 7]. As a result, the role of less well‐defined aggregates [8] and the loss of native proteins to the process of aggregation have been overlooked. However, the gain of amyloid and the loss of native protein occur simultaneously. Focusing solely on amyloid in discussions of protein dyshomeostasis risks presenting a one‐sided view—one that is, at best, incomplete and, at worst, misleading.

An epidemiological consequence of an amyloid‐centric definition of disease is that it forms the basis of a marked increase in the estimated prevalence of Alzheimer's disease (AD) over time, from ∼50 million cases to an expected ∼150 million cases by 2050 [9]. In fact, it is stated that “preclinical and prodromal AD may be more prevalent today than previously anticipated” [10] because, based on positron emission tomography (PET) data, amyloid prevalence rises with age among people with normal cognition: 17% between the ages of 50–54, a third by age 70, and more than half by age 95 [10]. However, while a simple majority of the population meets the “biological definition of AD” by the age of 85 (∼60%), based on amyloid‐PET positivity, only one‐fifth of them have dementia [11]. This review will seek to explain this paradox.

## 3 Gain vs. Loss

The field of neurodegeneration has been dominated by a conceptual framework around *gain*—of toxic proteins. Here, we argue that the *loss* of neurons and proteins are the most robust features of neurodegeneration. For instance, compared to healthy controls, the levels of alpha‐synuclein (α‐syn) in cerebrospinal fluid (CSF) are low in Parkinson's disease (PD) [12, 13], and the levels of 42‐amino acid amyloid‐beta (Aβ42) are low in AD [14]. Soluble proteins deplete while their insoluble state, *pathology*, becomes measurable and interpretable as *increasing*. What “goes up” are the markers of active neuronal loss, such as total tau (t‐tau), phosphorylated tau (p‐tau), and neurofilament light chain (NfL) [15, 16].

**FIGURE 1:**
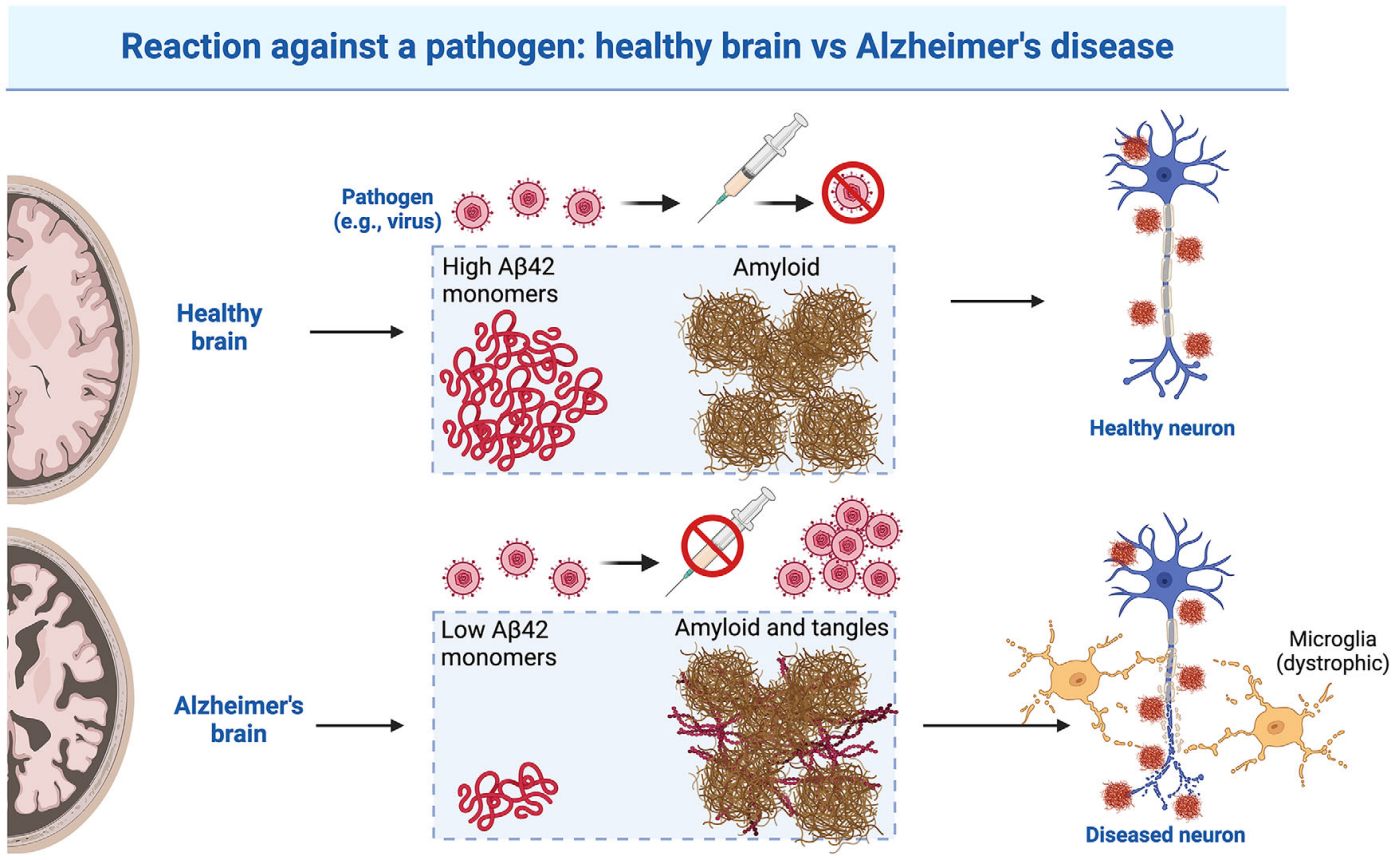
A reactive brain in health and disease. An external nucleation surface (such as that provided by herpes simplex virus type 1, in this example) catalyzes the reactive precipitation (polymerization) of normal monomers of Aβ42 into amyloid. Treatment against the pathogen (an antiviral in this example) eliminates the catalyst, preventing further polymerization of Aβ42, and maintaining high levels of the peptide. Delayed or lack of treatment perpetuates the polymerization and depletes the pool of polypeptides, potentially depriving neurons of crucial protein function and causing degeneration. Figure was created using Biorender tools.

Pathology originates from what was once a normal, soluble, monomeric protein. Upon a particular exposure—which varies across individuals and whose nature has largely remained invisible in brain aging research (but should be a future target for precision medicine)—normal proteins transform into abnormal, insoluble amyloid, adopting a universal cross‐β‐pleated conformation known as pathology. As pathology “goes up,” the soluble fraction of the original protein goes down. That is, the rise in pathology is relative, not absolute. Amyloid represents the insoluble fraction of the monomeric protein that is no longer present and whose functions become, therefore, lost. In this framework, a healthy brain may harbor substantial amounts of amyloid if monomeric protein levels remain normal, whereas a diseased brain will have low monomeric protein levels—regardless of the amount of amyloid present (Figure 1).

This hypothesis is particularly useful when trying to explain several apparent paradoxes. Epidemiological findings appear paradoxical when pathology presence or quantification is viewed in the absence of data on the levels of soluble monomers, the other end of protein homeostasis. It is paradoxical that half of centenarians with amyloid and tau pathology never develop AD [17, 18], and one‐fourth of them accrue α‐synuclein pathology without parkinsonism [19]. It is paradoxical that *increased* AD pathology can be associated with better cognition in healthy people [20] and patients with PD [21]. It is paradoxical that *increased* PD pathology in the substantia nigra is associated with more, not fewer neurons in the dopamine‐producing substantia nigra [22]. Pooling data from the largest study of centenarians [23], the odds ratio of dementia with AD pathology is 0.3 (95% confidence interval, 0.1–0.9) [24]. This suggests that forming “pathology” is not the cause of these diseases, and might even be a protective response from neurons, contributing to the ability to live beyond an average lifespan [24]. Lastly, it is paradoxical that by the ripe age of 85 years, 60% of us will have measurable amyloid in our brains, yet only 1 in 6 will develop dementia [11].

Several explanations have been suggested for these paradoxes. Most of them assume that unmeasured effects may come from the co‐occurrence of other pathologies or comorbidities (e.g., in [4]), or from what has been termed “cognitive reserve” or “resilience” (e.g., in [6] and [7]). Instead, we propose that these paradoxes can be resolved when both ends of protein homeostasis are considered. In AD, higher CSF Aβ42 levels are associated with normal cognition at any level of brain amyloidosis [25], even in amyloid‐positive carriers of AD‐causing genes [26]. Although we cannot yet measure the amyloid state of α‐synuclein in PD patients by PET, higher CSF α‐synuclein levels are associated with the preservation of normal brain volume [27], whereas lower α‐synuclein levels with brain atrophy and faster progression [27, 28].

### 3.1 Description of Pathology Spread

Data from brain autopsy studies, interpreted without the viewpoint of physics, have suggested that pathology “spreads” from one region to another. This was the influential description of Heiko Braak and colleagues on about 41 brains of unrelated individuals with a history of PD and 69 brains with PD‐defining pathology but from individuals without any such history [29]. Static data were interpreted as dynamic, with pathology *actively propagating* from one region to another. However, even within the Braak et al. dataset, there was no correlation between the density of pathology and cell loss, nor between the Braak stage and age, nor between the Braak stage and the clinical stage (Hoehn and Yahr) [30].

**FIGURE 2:**
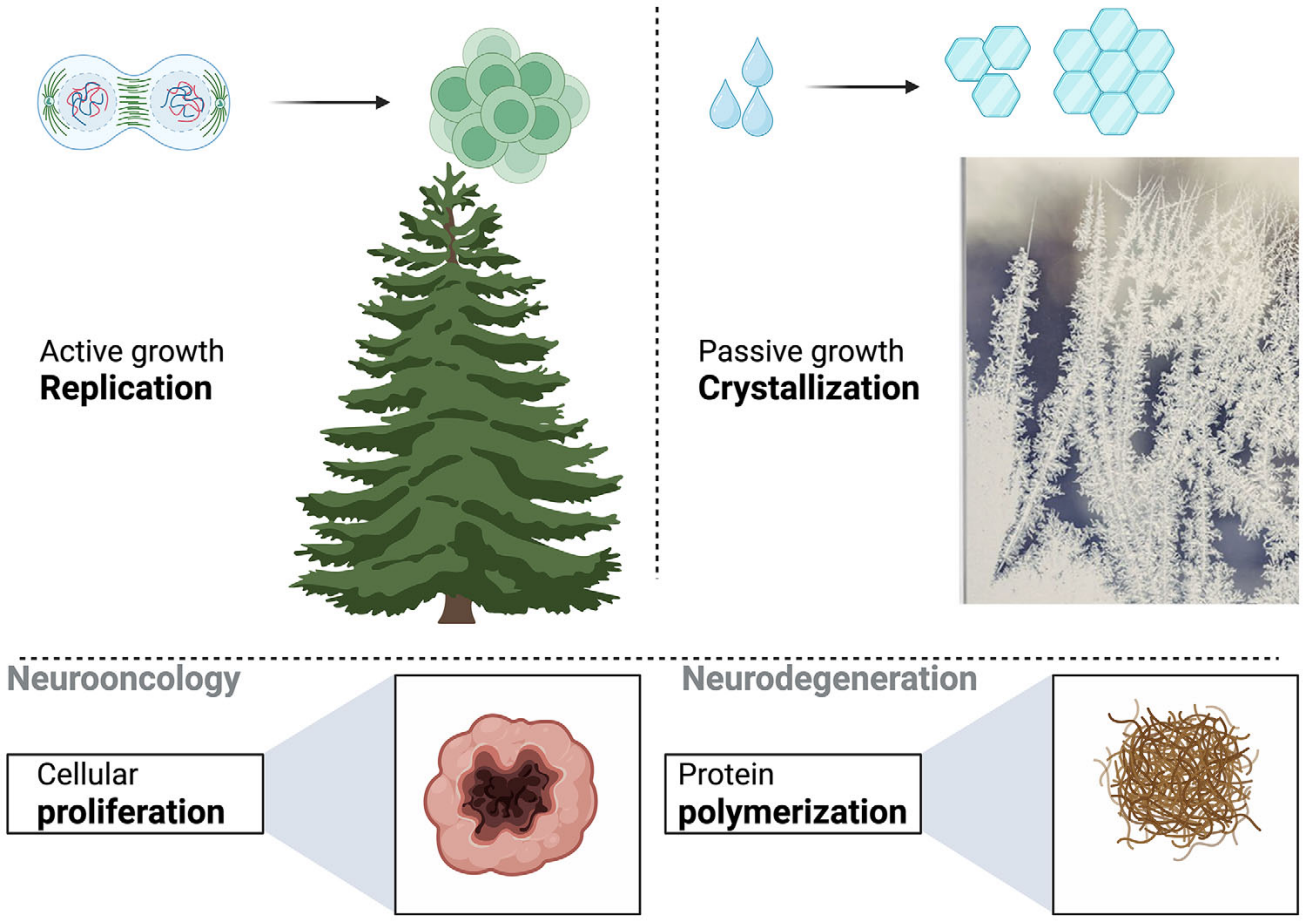
Active vs. passive growth. The growth of living organisms is an active process that depends on replication (nucleic acid required); the growth of non‐living matter can be passive, and does not require machinery for replication—as, for example, the spread of ice on a window during the winter. The phase transition of water into ice is an example of passive “growth” via crystallization. The growth or “spread” of pathology in neurodegenerative disorders reflects the transition of normal proteins without net gain (pathology is the relative insoluble fraction of former soluble monomeric precursors), unlike the net gain of cancers. Figure was created using Biorender tools.

Despite appearing similar on the surface, there are distinct types of “spread” or growth. The growth of living organisms is an active process that requires replication, whereas the growth of non‐living matter is passive and does not require replication (Figure 2). For example, plant growth relies on replication mechanisms, while crystal growth relies on phase transition phenomena that include several crucial steps such as nucleation and the increase of total concentration to reach the point of supersaturation (details below). Amyloid formation has been described as a form of the former (biological replication), yet this process should be explained using biophysical principles as a variant of the latter (phase transition). This passive “spread,” which will be discussed next, differs significantly from DNA‐based biological replication in terms of mechanisms and complexity.

## 4 Protein Aggregation From the Prism of Physics

To understand how this mechanism of “passive spread” works, it is necessary to introduce the concept of *thermodynamic equilibrium* and how it is related to protein structure. Thermodynamic equilibrium refers to a specific state in which a system's free energy is minimized. Changes in concentration and temperature can modify the equilibrium state of a system, sometimes resulting in a collective shift known as a phase transition.

For proteins, their native structure usually corresponds to their equilibrium state. Their stability arises from a balance between the favorable interactions that stabilize the folded state and the unfavorable entropic cost of folding [31]. Common initial folding patterns of a polypeptide chain (α‐helix and β‐sheet) are created from the hydrogen bonding between the N – H and C = O groups in the polypeptide backbone, and can, in principle, be formed by most proteins, regardless of their amino acid sequences.

**FIGURE 3:**
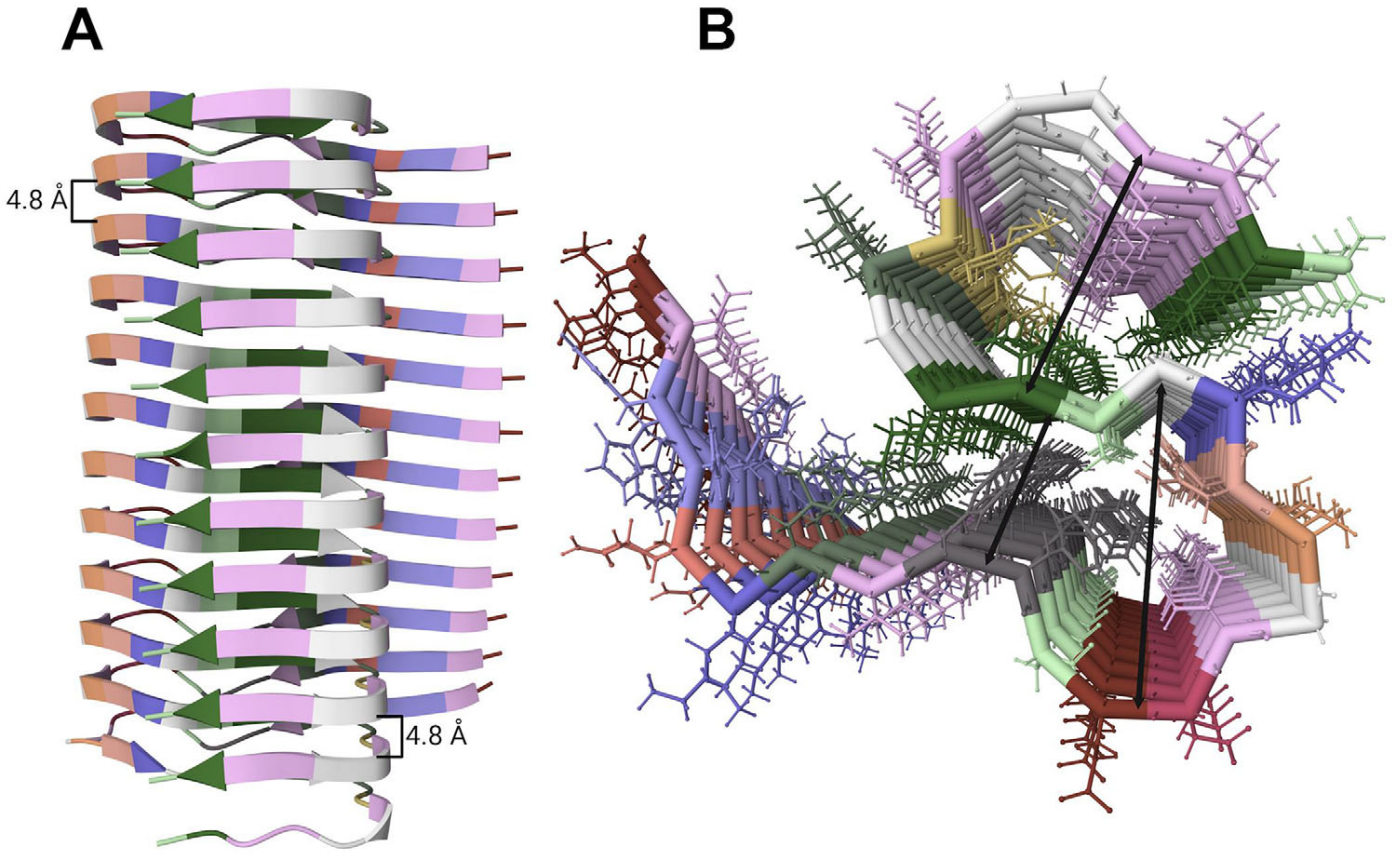
Amyloid structure. (A) Side‐view of a protofilament comprising multiple layers of monomers of the 42‐amino acid residue of amyloid‐β (Aβ42) separated by 4.8 Å. The amino acids (stripe colors) are stacked on top of each other in the common parallel in‐register arrangement. (B) Top‐view of the same protofilament. Arrows denote different cross‐β steric zippers between different stretched sequences of the peptide with interacting sidechains. Images created using Mol* [159] from PDB structure 2MXU from a paper by Xiao et al. 2015 [160].

Increasing the protein concentration reduces the entropy loss associated with aggregation, making the aggregated states more favorable [32, 33, 34]. At high enough concentration, proteins undergo a phase transition from a monomeric, soluble state to a multimeric, solid state. To achieve this, proteins typically adopt a fibrillar morphology. In this process, their native three‐dimensional structure flattens into a universal, thermodynamically stable cross‐β‐sheet configuration, forming amyloid or amyloid fibrils [35]. Amyloids arise from the stacking of cross‐β sheets, leading to the precipitation of formerly soluble proteins into an abnormal “solid” state (Figure 3). In contrast to the native protein conformation, which is uniquely defined by the amino acid sequence, β‐sheet stacking can be formed by any protein (including myoglobin [36], albumin [37], and insulin [38], as well as simple polyglutamate, polylysine, and polythreonine sequences) [39]. In fact, within the cytosol, proteins may form a dense phase non‐limited by any scaffold or a membrane, often referred to as biomolecular condensates, whose size can range from several hundred nanometers to a few micrometers [40]. The distinct electrical and chemical environments both within and at the interface of condensates [41, 42] can exacerbate protein oligomerization and fibrillation [43, 44, 45]. *Thus, the potential for proteins to fold into amyloid is dependent on the conditions of the microenvironment within which they reside* [46, 47, 48].

### 4.1 Thermodynamic Forces in Protein Transformation

According to what is known as Anfinsen's dogma, all the information required by proteins to adopt their native conformation is encoded in the amino acid sequence and this process “is driven entirely by the free energy of the conformation that is gained in going to the stable, native structure” [49]. In other words, adopting a certain protein conformation is a spontaneous process driven by the laws of equilibrium thermodynamics acting on the primary sequence of the protein in the physiological environment and requires no additional layer of information or a separate template [50]. Of course, this picture can be nuanced, as we know now that active processes control, repair, or help proteins fold in their native state [51]. Nonetheless, equilibrium thermodynamic is enough to explain the transition of a solution of proteins from their native state into amyloid fibers.

### 4.2 Thermodynamic Barrier and Nucleation

**FIGURE 4:**
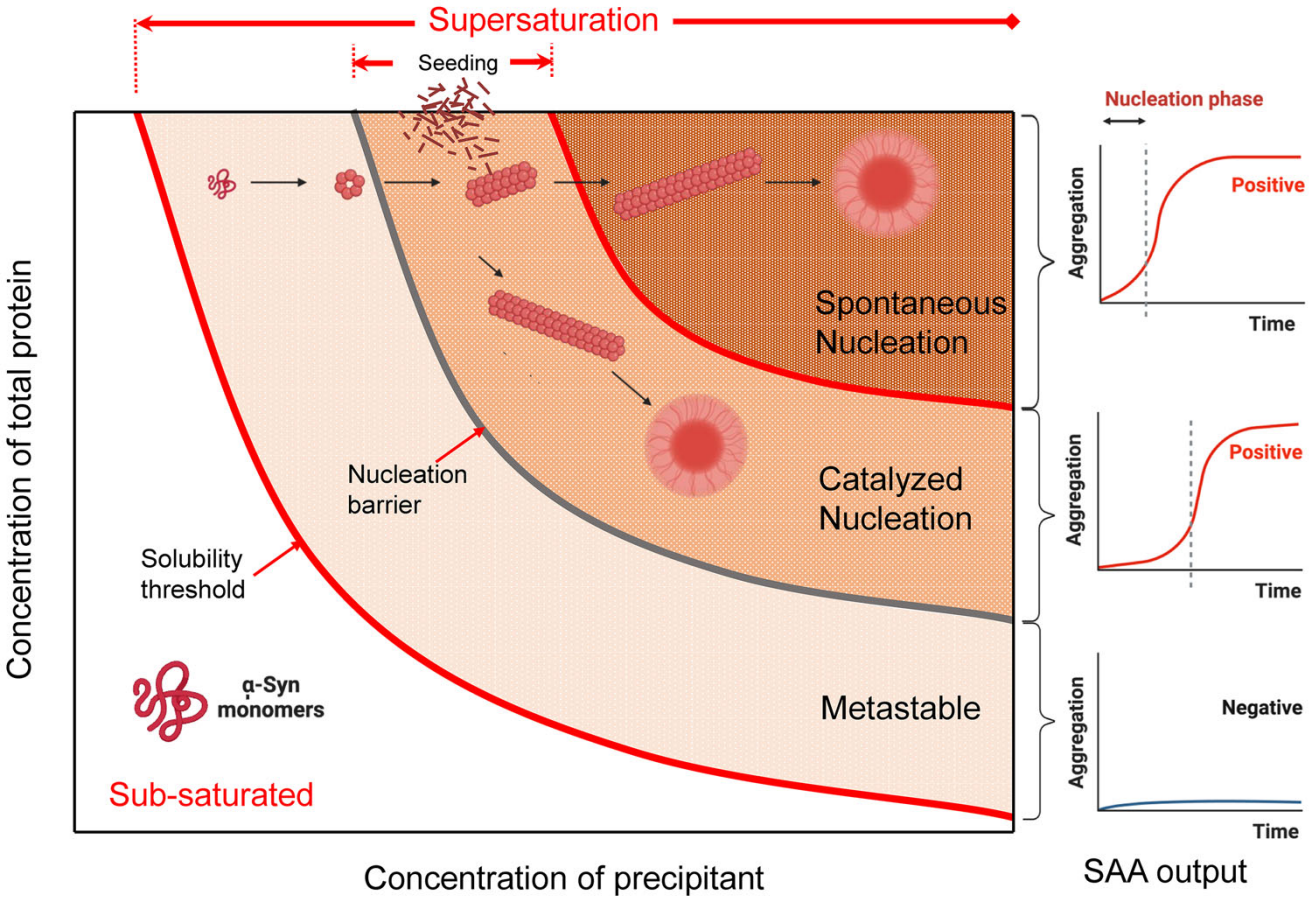
Supersaturation and nucleation. Below the solubility threshold, α‐synuclein monomers are natively folded in their thermodynamically stable state. In the intermediate energetic state (metastable), there is no spontaneous nucleation without a catalyzed lowering of the nucleation barrier by adding seeds (preformed nuclei) or heterogenous surfaces (viruses, lipid membranes, nanoparticles). Seeds catalyze the nucleation phase without a lag time (the experimental condition in the αSyn‐seed amplification assay [αSyn‐SAA]). With more significant supersaturation, the nucleation barrier is overcome, and nucleation proceeds spontaneously after a lag time. SAA figures in the right panel were created using Biorender tools.

The aforementioned transition requires crossing an energetically unfavorable state that represents a *thermodynamic barrier* (Figure 4) (Box 2) [37, 52]. This energetic barrier comes from two contributions. First, natively folded proteins have to partially unfold before creating a cross‐β aggregate. Second, forming the first piece of solid amyloid, the *nucleus*, also requires breaking the bonds between water molecules in the bulk of the solution to create a new interface between the amyloid solid phase and water (*nucleation*) [53].

**BOX 2. Amyloid nucleation via heterogeneous nucleation**

According to classical nucleation theory, the equation that describes heterogeneous nucleation is as follows:

 
$\Delta G^{HEN}=\Delta H_{amyl}-T\left(\Delta S_{protein\;}+\Delta S_{solvent}\right)+\left(4\pi \;r^{2}\;\sigma \;\times \frac{\;2-3\cos\theta +\mathrm{co}s^{3\;}\theta \;}{4}\right)$ where *r* is the radius of the nucleus, *σ* is the surface tension of the interface between the nucleus and the solvent, and θ is the wetting angle between the protein and the surface. The higher the affinity of the protein to the surface, the lower the wetting angle 𝜃, and the lower the nucleation barrier. In this regard, numerous interfaces have been shown to induce amyloid nucleation of proteins via heterogeneous nucleation.

**FIGURE 5:**
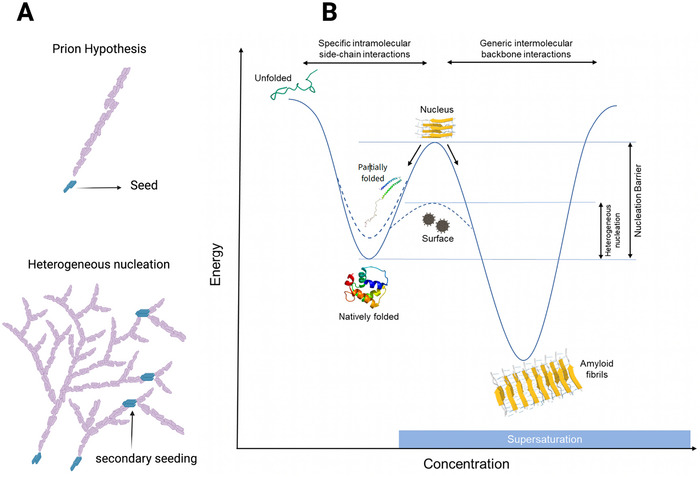
Native vs. amyloid protein folding and heterogeneous nucleation. (A) Amyloids grow like dendritic crystals, mediated by primary or secondary nucleation on the surface of a preformed nucleus (*seed*). There is no energy source or mechanism by which the seed can restrict growth to the tips, as proposed by the prion hypothesis. (B) Proteins assume their thermodynamically favorable (lower energy) native conformation based on specific interactions between the sidechains of their primary sequence under sub‐saturated conditions. Under super‐saturated conditions, greater molecular proximity makes unspecific intermolecular interactions more favorable. Partially folded proteins occupy a higher energy state than fully folded proteins and have a reduced nucleation barrier for aggregation. Preformed nuclei (seeding) or heterogeneous surfaces (heterogeneous nucleation) lowers the nucleation barrier, catalyzing the phase transition into amyloids. Heterogeneous surfaces may have exogenous (e.g., virus particles) or endogenous origins (e.g., disrupted sphingolipid membrane composition in *GBA* gene variants). Modified with permission from Ezzat et al. [73]. Figure was created using Biorender tools.

It is possible to lower the nucleation barrier by adding seeds (preformed nuclei) or catalysis surfaces. In the cell, lipids, proteins, and non‐proteinaceous factors might play such a role [54, 55, 56, 57, 58, 59, 60]. For example, the viral surface of herpes simplex virus type 1 (HSV‐1) acts as an exogenous nucleation surface and induces amyloid formation [61], depleting the corresponding soluble proteins [62], providing a plausible mechanistic link between latent/reactivating brain infections and neurodegenerative diseases [62, 63]. Separately, traumatic brain injury provides lipids and membranes that serve as endogenous nucleating surfaces [64]. Heterozygous variants in the *GBA* gene, a major genetic risk factor for PD [65], may promote α‐synuclein aggregation by disrupting lipid membrane composition [57]. Fibers arising from *seeding* and *heterogeneous nucleation* can grow by elongation, but also by *secondary nucleation*, where the fibers themselves act as nucleation surfaces for further heterogeneous nucleation [66, 67], and by *cross seeding*, where seeds of one protein can catalyze the amyloid formation of another protein just by acting as a surface [68] (Figure 5A).

### 4.3 Supersaturation

A solution is said to be supersaturated when the concentration and temperature conditions are such that the crystal is in a thermodynamically stable state. High concentration promotes molecular proximity, which makes nonspecific intermolecular interactions more likely [69]. However, the nucleation of the crystal requires overcoming a barrier that cannot be crossed spontaneously by the system. As a consequence, without the addition of a catalyst, or a crystal seed, the solution remains in its liquid, metastable, state. This is especially true for natively folded proteins, where the energetic barrier that needs to be reached in order to aggregate is tied to the stability of the native state. In contrast, disordered or mutated proteins, with less stable native conformations, are more prone to aggregation (Figure 5B) [70, 71].

### 4.4 Amyloid Formation

As discussed in this perspective, the formation of compact and ordered amyloid fibers from proteins in solution is called a *phase transition* [52, 72]. Just as water can exist in different states or phases of solubility depending on their packing density (vapor, liquid, or ice), proteins can also be soluble in a liquid microenvironment, condensed in a liquid‐liquid phase separation, or tightly packed in a solid‐like state. In this context, amyloid formation occurs through a process of thermodynamic equilibrium, similar to protein folding under the hypothesis of Anfinsen [73]. This process, sometimes wrongfully referred to as *polymerization* because it does not involve the breakage or formation of covalent bonding, is in fact, a process of *crystallization*. In principle, changing the thermodynamic conditions should revert the process, yet, just like a diamond, a crystal, it may remain stable under unfavorable thermodynamic conditions [74]. This process involves three types of changes: *structural*, from natively folded to a cross‐β; *physical*, from soluble to insoluble; and *biological*, from functional to non‐functional [75]. The ongoing formation of amyloid is more dependent on the “growth” conditions (pH, buffer, shaking, temperature and presence of nucleation catalyst) rather than the two‐dimensional‐fold morphology of the amyloid fibers (seeds) [76, 77].

Importantly, it has been assumed that the two‐dimensional structure of amyloid fibers should be conserved in patients with the same disease. However, studies proposing the within‐disease architectural consistency of fibers have largely relied on single cases (Box 3). Analyses of brain tissues of two [78] or three individuals [77] with the same disease, multiple system atrophy (MSA), show that the architecture of the amyloid structure differs between patients (Panels B and C in Figure 3 of the manuscript by Sawaya et al., “A single protein sequence attains multiple amyloid polymorph structures”) [78] or cannot be replicated with fidelity when combining recombinant α‐synuclein with the seeds from the putamen of three different patients with the same disease [77]. This further supports the concept that in vitro conditions, rather than the morphology of the seeds determine the structures formed in the phase transition from the monomeric to the amyloid state, resulting in the creation of polymorphs (variable two‐dimensional folds), not strains (identical copies) (Table 1).

**TABLE 1:** Replication vs. nucleation (amyloid phase transition).

	Replication	Nucleation
Process	**Active**, ATP‐dependent	**Passive**, dependent on concentration, temperature, pH, and surface catalysts
Mechanism	**Double helix**—Pairing mechanism (A:T, C:G) ensures replication with fidelity	**Supersaturation**, which makes intermolecular bonds more thermodynamically favorable
Output	**Exact copy**, independent of microenvironment	**Stochastic growth**, dependent on microenvironment
	***Strains*** *—*Identical copies can be faithfully replicated	***Polymorphs***—Generic fibrillar structure

**BOX 3. Can specific amyloid folds (pathology) be disease‐specific?**

Recent studies (e.g., Shi et al., Nature 2021) [79] have suggested that specific two‐dimensional folds of pathological amyloid, as visualized by cryo‐electron microscopy, might be disease‐specific. However, these findings should be interpreted with caution:

1. Most of these studies (e.g., an atlas of tau filament folds to classify tauopathies [79]) do not report statistical associations and are often based on single cases (*n* = 1).
2. Amyloid polymorphs are highly sensitive to the conditions under which they are harvested. Factors such as pH, ionic strength, and agitation—all of which can be altered during centrifugation—can selectively enrich a given set of polymorphic folds.
3. Comparing polymorphs obtained from different brain areas, at different disease stages, or harvested by different methods will inevitably lead to polymorphic differences that cannot be reliably linked to clinical differences, especially in the absence of standardized methods and statistical controls.
4. Despite surface differences, all polymorphs share the same core cross‐β conformation, which dictates their fibrillar protofilament shape and underpins their fundamental properties of stability and minimal reactivity.
5. Lastly, it is unclear why subtle differences in fibril shape would lead to dramatic differences in disease phenotypes.

In sum, without statistics, standardized methodology or mechanistic insight, clinical correlations or classifications based on amyloid polymorphs are unwarranted.

To summarize, thermodynamically favorable conditions are necessary, but not sufficient for amyloids to nucleate and grow. In the absence of seeds or catalysis, a solution may remain in a super‐saturated state forever. When amyloid starts to form, its propagation induces a lowering of the concentration of the native protein, until the solution becomes sub‐saturated and the phase transition/crystallization ends. It is primarily the thermodynamic conditions (concentration, temperature, seeds), rather than the amino acid sequence or structure of the resulting amyloid, that are the deciding factors of whether a protein adopts a native or cross‐β amyloid conformation [73]. A protein adopts its native conformation at physiologic concentrations and its amyloid conformation under conditions of supersaturation and the availability of a nucleating agent.

## 5 A Biophysical Framework for Interpreting Neuropathology

Describing the generation of pathology in biophysical terms, accounting for both the soluble and insoluble ends of protein homeostasis, helps resolve apparent paradoxes. From this perspective, pathology consists of a cross‐β structure, one of the most stable configurations in nature and, therefore, unlikely to be toxic [80], generated through a crystallization‐like process [81, 82].

A biophysical reconsideration of neurodegeneration might begin with a few broadly accepted concepts: (1) proteins have functions; (2) proteins typically require their native conformation to function properly; (3) when proteins lose their native conformation, their ability to function is impaired; and (4) as a result, the loss of normal proteins to amyloid states during aggregation may have important functional consequences. The implications of these ideas could be significant. In contrast to the well‐established toxic effects of high serum cholesterol or high blood glucose, many individuals with AD or PD are found to have low levels of cerebrospinal fluid Aβ42 or α‐synuclein. Thus, the pathological state of proteins represents the relative “gain” of insoluble monomeric proteins lost to aggregation.

A biophysical framework to explain neurodegeneration stands in contrast with the century‐old clinicopathologic framework (Table 2).

**TABLE 2:** Clinicopathologic vs. bio‐physical neurodegeneration frameworks.

	Clinicopathologic framework	Biophysical framework
**Pathology (amyloid)**	Cause	Effect
**Pathogenesis**	Accumulation of amyloid	Depletion of monomeric protein
**Conformation**	Strains	Polymorphs
**Pathology spread**	Active, Replication	Passive, Nucleation
**Treatment approach**	Amyloid clearance	Monomeric protein restoration

### 5.1 Are Amyloids Toxic?

The cross‐β structure of amyloids gives them stability and insolubility, which results in low reactivity [73]. These characteristics make amyloids less likely to inflict toxicity through biochemical reactions. However, the transition from soluble to insoluble forms decreases the concentration of functional proteins in solution. As the levels of soluble proteins drop, their ability to perform essential cellular functions diminishes. Therefore, while amyloids themselves are not inherently toxic, the process leading to their formation depletes normal functional proteins, which disrupts cellular activities [83]. Remarkably, the recognition that a mechanism for a gain of toxic function from amyloids “remains largely unknown” has not detracted from efforts at defining its atomic structure for “the development of more specific and safer treatments” [84].

### 5.2 Are Amyloids Polymorphs or Strains?

This accessibility of the cross‐β conformation to most peptide sequences leads to the phenomenon of polymorphism, where different sequence stretches within a protein prefer different ladder stacking and mating orientations depending on environmental conditions during amyloid formation. Thus, a single protein sequence can adopt different two‐dimensional bends, curves and folds to accommodate different ladder and zipper arrangements (Box 4) depending on pH, salt concentration, temperature, or even shaking [78]. Such environmentally sensitive polymorphism results in different cross‐sectional shapes of the protofilament. Furthermore, protofilaments can twist and associate in different configurations, leading to further polymorphism at the fibrillar level [85].

**BOX 4. Ladder and zipper in protofilament formation preceding amyloids**

Amyloids form β‐sheet ladders, comprising thousands of molecules extending over micrometers [35, 78, 86]. These opposing ladders interdigitate like zipper teeth, expelling water to create a “dry steric zipper,” which enhances amyloid formation [87]. Ladder pairing is based on the chemical complementarity of the side chains of each ladder. For example, hydrophobic or polar side chains tend to mate with counterparts of similar properties, and ladders of positively charged amino acids tend to mate with ladders of negatively charged ones [78]. Protein monomers are spaced 4.8 Å within ladders, while opposing β‐sheets are 6–12 Å apart, producing a characteristic amyloid x‐ray diffraction pattern. Monomers stack in parallel, though exceptions for antiparallel alignment exist [88]. Interdigitating β‐sheet pairs form protofilaments, the fundamental amyloid subunit, which further assemble into fibrils and eventually amyloid plaques.

*Strain* refers to a distinct variant or subtype of a microorganism and has most often been applied to a “genetically stable virus variant that differs from a natural reference virus in that it causes a significantly different, observable, phenotype” [89]. In neurology, “strains” have been used for protofilament or fibrillar polymorphs of distinct morphology that can “template” the formation of amyloid with fidelity via serial elongation at the seed fibril ends [90]. Not only has the stability of different pathology conformations not been established, but the structures of the fibers have been documented to differ from those of the seeds from which they come [77], with their folds varying according to the microenvironmental conditions of the tissue rather than the configuration of the seed [76]. These local environmental conditions decide the most favorable β‐strand ladder stacking and steric zipper mating within the cross‐β amyloid architecture, which can lead to a variety of two‐dimensional‐folds and superstructural morphologies (different numbers of protofilaments per fibril, flat vs. twisted fibrils) [78]. External factors, such as inorganic salts and mechanical agitation, also influence the ultrastructural shape of filaments, as demonstrated in tau amyloid structures [91]. This diversity aligns with equilibrium thermodynamics, where different crystalline arrangements are classified as allotropes. Ice, for instance, has nineteen known crystalline arrangements. As with simple crystals, changes in thermodynamic conditions can induce the transformation of one allotrope into another through a phase transition. The more complex the molecular interactions are, the larger the diversity of allotropes. This process involves nucleation followed by crystal growth. In some cases, an allotrope may remain in a metastable state, leading to a heterogeneous population of fibril structures.

The in vitro observations noted above support the conclusion that environmental conditions influence the characteristics of the product of aggregation but are not useful to provide a mechanistic perspective for any particular disease entity. As opposed to the purely replicative mechanism proposed for prions, the physical framework of nucleation and growth is compatible with the generation of polymorphic (rather than structurally identical) fibers. The structure of polymorphs highly depends on the microenvironment, not the structure or morphology of the seed [75]. The strain‐like quality for prions has only been supported using such laboratory tools as differential resistance to disaggregation and proteolysis [92].

## 6 Physical Basis to Reinterpret Common Statements About Protein Aggregation

### 6.1 Amyloid Plaques Sequester Neurotoxins and Microbes

Next to its proposed role as a cause of AD, amyloid formation is also conceived as a way to “sequester oligomers in a non‐diffusible, less neurotoxic state” [93], according to the amyloid cascade hypothesis proponents; or, more generally, as a “sequestration process whereby the brain seeks to attenuate neurotoxicity” [94], according to prion hypothesis proponents; or, in the case of brain infections, as “an extracellular trap for the microbe” [95], according to antimicrobial protection hypothesis proponents. The contradiction that amyloid may be conceived both as an agent of toxicity and a mechanism for toxin sequestration or “antimicrobial activity” [96] can be resolved by applying biophysical principles. The heterogeneous surfaces of toxic nanoparticles or viral protein coronas lower the thermodynamic barrier for amyloid formation by catalyzing the transition of soluble Aβ into insoluble amyloid [97]. Thus, in infections with neurotropic viruses like HSV‐1 and human herpesvirus 6A and B, amyloid formation represents a protective response [98], rather than the agent of toxicity.

### 6.2 Mutation Carriers Have High Levels of Aβ42

Both *APP* duplication and *SNCA* duplication, two of the most common genetic etiologies of AD and PD, respectively, are traditionally viewed as disorders “overexpressing” amyloid. This supports the assumption that mutation carriers should have higher levels of soluble Aβ42 compared to non‐mutation populations. However, mutation carriers have *lower* Aβ42 levels than non‐mutation age‐matched populations [99]. The reduction in soluble Aβ42 levels among mutation carriers starts as early as 25 years before the onset of cognitive symptoms [100]. The biophysical mechanism behind these genetic abnormalities involves protein supersaturation: the increased production of Aβ42 supersaturates the solution, promoting the nucleation process, which accelerates amyloid formation at the cost of depleting the substrate. Thus, just like in sporadic AD, soluble Aβ42 is depleted in familial forms of the disease.

#### 6.2.1 The Special Case of Supersaturation in Genetic Mutations

Genetic mutations can also increase vulnerability by making a protein unstable, less soluble, or overexpressed. This is the case in many pathogenic mutations that cause neurodegenerative diseases. For example, the H50Q mutation in α‐synuclein, encoded by the *SNCA* gene, results in a 10‐fold decrease in its solubility, increasing α‐synuclein supersaturation and its propensity to aggregate [101]. Gene duplications of *APP*, as in some cases of familial AD and Down's syndrome, and of *SNCA*, such as in familial PD, will also lead to higher supersaturation and lower the energy barrier for aggregation. With increasing amyloid formation there is consequent protein consumption, ultimately leading to low levels of soluble protein monomers of Aβ [99, 102], and α‐synuclein [103].

#### 6.2.2 Resolving the Amyloid Paradox

If dementia is invariably associated with low Aβ42 levels but poorly associated with high amyloid levels, do higher levels of soluble Aβ42 predict normal cognitive function better than lower levels of brain amyloid in amyloid‐positive individuals? Over an observation period of 3 years among carriers of AD‐causing mutations in *APP*, *PSEN1*, or *PSEN2*, we found that *higher* levels of soluble Aβ42 predicted a lower risk of cognitive impairment to a greater extent than lower levels of brain amyloid [26]. CSF Aβ42 levels > 270 pg/mL predicted a reduced risk of cognitive progression *regardless of increases in brain amyloid*. Higher levels of soluble Aβ42 were also associated with larger hippocampal volume and normal brain metabolism in the precuneus. Supporting the importance of the soluble protein fraction, individuals with dementia associated with the rare *APP* E693del (Osaka) mutation exhibit low soluble Aβ42 levels despite having no brain amyloid [104]. Cases with brain amyloid/soluble levels (PET/CSF) discordance are informative: the frequency of AD is significantly higher in PET−/CSF+ (low concentration of CSF Aβ42 in the absence of amyloid) than in PET+/CSF− [105, 106], suggesting that brain amyloid without a reduction in soluble Aβ42 levels is unlikely to be associated with dementia.

### 6.3 Ratios Are Better Than Absolute Levels

Because Aβ40, the peptide species with two fewer amino acids, is less functional than Aβ42 and has a lower capacity to aggregate (the presence of residues 41 and 42 in Aβ42 plays an important role in lowering the energy barrier for the transition of monomer to fibrils) [107], it has been used to create a benchmark against which to understand the variations of Aβ42 [108]. Two early studies noted that using Aβ42 and Aβ40 together in a ratio as Aβ40/Aβ42 [109] or the inverse Aβ42/Aβ40 [110] classified a population of clinically‐defined AD patients slightly (non‐significantly) better than the levels of Aβ42 alone. The decrease in Aβ42/Aβ40 or increase in Aβ40/Aβ42 is due to the much lower decrease of Aβ40 relative to the marked decrease of Aβ42 as it nucleates into amyloid. While a panel of experts has recommended using the Aβ42/Aβ40 ratio over Aβ42 levels alone [111], Aβ42 levels show the highest diagnostic performance for AD in an autopsy‐confirmed cohort, whereas Aβ40 is the lowest [112], which may explain the lack of correlation between the Aβ42/Aβ40 ratio and the age of onset in mutation carriers [113]. More importantly, from a biophysical standpoint, amyloid aggregation is dependent on supersaturation [114, 115, 116], which is based on the absolute concentration of a peptide, irrespective of its relative levels compared to other peptides. Decreasing the absolute concentration of a peptide reduces the saturation and the related propensity to aggregate [73].

### 6.4 Loss of Soluble Aβ42 Is Inconsequential

A protein can only function when in its native, soluble state, and ceases to function once transitioned into amyloid. A reduction in absolute levels of CSF Aβ42 is associated with brain atrophy in cognitively normal elderly [117]. This suggests that soluble Aβ42 is important for normal brain function, and its loss is consequential. In fact, at physiological (picomolar) concentrations, Aβ42 modulates synaptic function and plasticity, facilitates neuronal growth and survival, reduces iron toxicity, and protect against oxidative stress and against toxins and pathogens [118, 119, 120, 121, 122, 123, 124, 125]. Suggesting a role as a neuropeptide, Aβ42 is stored in presynaptic dense core vesicles and is released upon neuronal stimulation [126], binds with high affinity to the α7 nicotinic acetylcholine receptor (α7‐nAChR), which is highly expressed in the hippocampus and cortex [127], induces its effects via modulation of glutamatergic transmission [128], and is catabolized by the action of an endopeptidase, neprilysin [129]. Importantly, it has been experimentally demonstrated that monomeric Aβ42 supplementation can rescue behavioral deficits both in PSEN1/PSEN2 conditional double knockout AD mice and in APP/PSEN/Tau triple transgenic AD mice, an effect mediated through binding of Aβ42 to α7‐nAChR [130].

### 6.5 Oligomers Are Toxic

The idea that Aβ oligomers are toxic emerged as an explanation for the unclear relationship between amyloid levels and disease progression. *If neither amyloids nor monomers cause degeneration, intermediate oligomers must be the source of the problem*. From a biophysical perspective, the conditions that allow amyloid fibril formation (supersaturation and nucleation) do not support the stable accumulation of oligomers. Instead, oligomers tend to either dissociate back into monomers or proceed to form fibrils [73]. This is supported by models of oligomerization and aggregation [131], which show that the concentration of oligomers decreases as aggregation progresses. The energy dynamics further explain this: when a dimer aggregates onto a fiber, the energetic benefit outweighs the loss of free movement, making aggregation more favorable. Finally, oligomers depend on a continuous supply of monomers, but Aβ42 monomers decrease as the disease progresses, reducing the likelihood of oligomer formation. A depleting protein species is unlikely to be the primary source of toxicity.

### 6.6 Proteins Propagate in a Prion‐Like Manner

A replicative or prion‐like effect of proteins was introduced by Stanley Prusiner, for which he was awarded the Nobel Prize of Medicine. Prusiner's prion hypothesis postulated that an “infectious” protein has the capacity to self‐propagate and be transmissible as distinct “strains”. Inoculation into naïve brains of amyloid‐containing brain material treated with ultraviolet light (UV) induced amyloid propagation and neurodegeneration [132]. This experiment led to the conclusion that since UV inactivates the nucleic acid of any virus, and a virus had been assumed to be required for the “propagation,” the agent of spread was a non‐viral but transmissible effect of protein, a prion [133]. Although never demonstrated in humans, several in‐vitro and in‐vivo studies supported a “prion‐like propagation” [134, 135]. Protein‐only prions (PrP) were hypothesized to carry the conformational information required to template the transformation of normal proteins into amyloids. However, HSV‐1 and respiratory syncytial virus, in the absence of a protein seed acting as a conformational template, can induce protein aggregation via heterogenous nucleation [97]. Also, UV‐inactivation does not prevent SARS‐CoV‐2 viral particles from catalyzing nucleation on their surfaces and inducing amyloid in human CSF [136]. Thus, the deactivation of viral nucleic acids does not affect the ability of viruses or other membranous structures to act as catalytic surfaces to induce amyloid aggregation. Despite the apparent similarities, the nucleation and growth mechanism can lead to a metastable state, in which the system does not always settle into its equilibrium state. Lastly, the PrP conformation believed to cause Creutzfeldt‐Jakob disease (CJD), known as PrP^Sc^ (“prion protein scrapie”), share the same cross‐β conformation visualized by x‐ray diffraction in Aβ42, α‐synuclein, TDP‐43, tau, and other aggregated proteins [85]. This is a generic conformation of any protein under supersaturated conditions. It is thus possible that the rather dramatic disease course of CJD is the result of the loss of the neurotrophic effects of the PrP in its normal, monomeric state [137, 138].

There is indeed evidence to support that PrP monomer loss to rapid fibril formation may be more relevant to CJD than the “cell‐to‐cell spreading” of its pathological form. First, PrP is highly conserved in mammals [139] and is expressed in most tissues of the body, most prominently in brain neurons [140]. Second, PrP has roles in copper‐binding [141], nucleic‐acid‐binding antimicrobial function [142], and in modulating a number of membrane receptors, especially glutamate, ion channels, and amino acid transporters [143]. Third, because PrP is concentrated in synaptic terminals, where it co‐localizes with synaptophysin, its transition into PrP^Sc^ contributes to synaptic dysfunction and loss [144, 145].

Notably, a key assumption to the templated seeding in the prion hypothesis is that the newly formed amyloids adopt the same structure as the seed. However, the only study to date examining both seed structures and their aggregated products—specifically α‐synuclein filaments from the putamen of three individuals with MSA used as recombinant in vitro seeds—found that the seed structures were not replicated [77]. This further supports the conclusion that pathology does not spread actively by producing structurally identical copies of a seed (also referred to as “strains”). Instead, it spreads passively through a phase transition mediated by primary and secondary nucleation, which depends on the cellular microenvironment rather than any specific amino acid sequence or amyloid fold (Table 1).

### 6.7 A Positive Seed Amplification Assay Means Brain Seeding

Seeding is one of three nucleation mechanisms by which proteins phase transition. A seed catalyzes the aggregation of peptides in layers around a seed in a process that is passive. The α‐synuclein seeding amplification assay (α‐Syn SAA), also known as real‐time quaking‐induced conversion (RT‐QuIC) or protein misfolding cyclic amplification (PMCA), has been increasingly used in PD research to identify the presence of tissue containing aggregated α‐synuclein. In these tests, the reagent is monomeric α‐synuclein, marked for detection with amyloid‐binding dye thioflavin T (ThT), at a concentration 1 000 000 higher than physiologic in CSF (that is, measured in µg/mL rather than pg/mL). This supersaturated reagent helps bypass the rate‐limiting nucleation step by providing the system with ready‐made nuclei, which can accelerate amyloid formation either via direct elongation on the tips of the seed or via surface‐catalyzed (heterogenous) nucleation on the seed surface of any aggregated α‐synuclein (nucleation catalyst) present in the tissue examined. The enhanced fluorescence of ThT allows the catalyzed amyloid formation to become detectable, making it a “positive” test. This α‐Syn SAA is excellent at detecting the presence of amyloid‐state protein in a tissue but, by virtue of the supraphysiologic conditions required in the laboratory, it is not a demonstration of pathogenesis in the human brain nor, as it is increasingly conceived, an identification of a particular biology in the individuals tested.

A biophysical approach avoids confusing “seeding” with “replicating” α‐synuclein. Under the conditions of the test, any “seeds” (aggregated protein) present in the tested tissue prefer, thermodynamically speaking, to be in a solid state rather than in a liquid state.

### 6.8 Amyloid as a Normal Feature of Aging

Many neurodegenerative “proteinopathies” share a common risk factor: advancing age [146]. The laws of physics as it relates to proteins apply to aging cells, which endure numerous external insults and detrimental changes in cellular function. As amyloid prevalence increases linearly with aging, amyloid formation could be a physiological response of the brain to maintain homeostasis against age‐related toxic or infectious exposures or biological abnormalities. Professor of Neurobiology Karl Herrup has argued that the relationship between amyloid and dementia is similar to that between gray hair and dementia: they precede it but do not cause it [147, 148]. Observations from brain banks show that most amyloid‐positive brains (brains containing multiple pathologies) come from people without a history of neurological problems [149, 150], and the match between pathology and disease only ranges from 19% to 45% [151]. This suggest that amyloid may be the hallmark of normal aging‐related brain reactivity. Pathology alone does not predict disease because, in most cases, the brainop is capable of maintaining a pool of monomeric precursors within a normal range. Only when these levels are too low in PD [28] or AD [25, 26], pathology is associated with the disease—an association dependent on the consumption of normal protein, not on the level of pathology.

## 7 A Biophysical Interpretation of Anti‐Amyloid Monoclonal Antibodies

The recent Food and Drug Administration approvals of the anti‐amyloid monoclonal antibodies aducanumab, lecanemab, and donanemab for the treatment of AD may give the impression that reducing amyloid is enough to slow cognitive decline, supporting the hypothesis that amyloid is toxic. However, these drugs show statistically but not clinically significant benefits. They lead not to clinical improvements but slower cognitive decline, at a magnitude that is half of what patients would be able to notice as “minimal clinically important,” while carrying significant risks: a 1‐in‐3 chance of harm, including a 1‐in‐4 chance of brain swelling and/or bleeding (potentially fatal but sanitized under the acronym “ARIA,” for “amyloid‐related imaging abnormalities”), and accelerated rate of brain atrophy [152, 153, 154, 155].

Still, even if the clinical effects are modest, why would removing amyloid have any benefit at all from a biophysical perspective? Unlike systemic amyloidosis affecting certain tissues (e.g., the myocardium), there is no direct evidence that brain amyloid exerts a mass‐occupying effect (volume‐based toxicity) or is associated with inflammation or degeneration on surrounding brain tissue (direct toxicity). As we have argued throughout this review, amyloid represents the end of the monomeric or native configuration of proteins, and therefore, the end of their functions, not the beginning of a toxin. What effect do anti‐amyloid monoclonal antibodies have on monomeric Aβ42 in clinical trials? They actually *increase* its levels [156]. In a recent analysis of nearly 26 000 patients participating across 24 randomized clinical trials of anti‐amyloid antibodies, we found that a similar magnitude of cognitive changes was predicted by the *increases* in CSF Aβ42 as by the reductions in brain amyloid [157]. Increasing CSF Aβ42 levels may be an overlooked but relevant mechanism by which anti‐amyloid monoclonal antibodies may exert a positive effect.

## 8 Conclusions and Outlook

The physics of protein aggregation in normal and accelerated brain aging are the same: proteins undergo phase transitions into their amyloid states, marking the end of their native—and therefore functional—configuration. In normal aging, monomeric peptides are replaced at a rate that matches their consumption into amyloids; in accelerated aging, this replacement is inadequate, leading to neurodegenerative disorders. In this review, we offer an alternative biophysical framework to the standard clinicopathological language about proteins. We suggest that PD, AD, and other neurodegenerative diseases are not caused by the accumulation of pathology; that proteins do not shift from physiologically important molecules to agents of disease (“proteinopathies”), or become “toxic strains” that “self‐replicate,” spreading in a “prion‐like” manner by “templating.” Instead, proteins continue to behave like proteins: they precipitate rather than replicate, they lose their function when abnormal, and they do not increase to toxic levels—instead, they deplete. With the loss of functional proteins comes the progressive atrophy of the human brain undergoing degeneration.

Aβ42 and α‐synuclein have been preserved across the animal kingdom, with little variation for at least 400 million years, suggesting they are critical for normal brain function. A century‐old clinicopathologic framework has emphasized that once these proteins convert into amyloid, they must become toxic and explain neurodegeneration. Replacing this descriptive framework with a biophysical one redefines pathology not as a toxin (or as a means to sequester ostensibly toxic oligomers), but as the result of the loss of soluble, functional protein due to supersaturation and catalytic nucleation. In this view, brain degeneration is not driven by the shape of amyloid fibrils (polymorphs), but by the depletion of normal monomeric proteins as they aggregate. Therefore, treatments that restore these proteins to their normal levels could help counteract the effects of biological, toxic, or infectious stressors that create the microenvironmental brain conditions that catalyze the phase transition of proteins from their native to the universal cross‐β configuration of their amyloid state. This rescue approach has already been demonstrated in two mice models of AD (PSEN1/PSEN2 conditional double knockout mice, without amyloid deposition, and APP/PS1/Tau triple transgenic mice, with amyloid deposition) [130] and in a rat model of PD (α‐synuclein knockdown) [158] but has yet to be tested in humans. If amyloid levels were to rise during the evaluation of such a treatment, this would likely reflect a faster monomeric‐to‐pathological protein conversion—suggesting the need for more frequent dosing. According to biophysical principles, more amyloid means less functional protein, not more toxicity.

In closing, we propose a shift in how brain aging is studied—from a clinicopathological model that treats pathological proteins as the drivers of neurodegeneration, to a biophysical model that emphasizes maintaining protein homeostasis. This reorientation reframes the treatment goal: instead of clearing amyloid, the focus becomes restoring monomeric protein levels above the threshold needed to sustain brain function.

## Author Contributions

A.J.E. drafted the manuscript. All other authors critically reviewed the manuscript.

## Conflicts of Interest

Prof. Alberto J. Espay has received grant support from the NIH and the Michael J Fox Foundation; personal compensation as a consultant/scientific advisory board member for Mitsubishi Tanabe Pharma America (formerly, Neuroderm), Amneal, Acorda, Abbvie, Bial, Kyowa Kirin, Supernus (formerly, USWorldMeds), NeuroDiagnostics, Inc (SYNAPS Dx), Intrance Medical Systems, Inc., Merz, Praxis Precision Medicines, Citrus Health, and Herantis Pharma; Data Safety Monitoring Board (chair) of AskBio; and publishing royalties from Lippincott Williams & Wilkins, Cambridge University Press, and Springer. He is co‐inventor of the patent “Compositions and methods for treatment and/or prophylaxis of proteinopathies.” He cofounded REGAIN Therapeutics to fund preclinical studies but relinquished the right to any personal income from future treatments. He serves on the editorial boards of the Journal of Parkinson's Disease, Journal of Alzheimer's Disease, European Journal of Neurology, Movement Disorders Clinical Practice, and JAMA Neurology. Andrea Sturchio cofounded REGAIN Therapeutics and is co‐inventor of the patent “Compositions and methods for treatment and/or prophylaxis of proteinopathies.” He has received personal compensation from Baillie Gifford. Alberto Imarisio, Emily J. Hill, Brady Williamson, Kora Montemagno, and Christian Hoffmann report no disclosures. Hugo Le Roy has received grant support from the EMBO Scientific Exchange Fellowship. Dragomir Milovanovic has received grant support from the German Research Foundation (MI 2104 and SFB1286/B10), the Human Frontier Science Program (RGEC32/2023), and the ERC Grant MemLessInterface (101078172). Fredric P. Manfredsson has received grant support from the NIH, DOD, the Parkinson Foundation, and the Michael J Fox Foundation; personal compensation as a consultant for Aspen Neuroscience, Seelos Therapeutics, Regenxbio and publishing royalties from Springer. He cofounded nVector, CavGene Therapeutics and Neuralina Therapeutics, and is co‐inventor of the patents “Nurr1 as a genetic target for treating levodopa‐induced dyskinesias in Parkinson's disease,” Systems and methods for treating levodopa‐induced dyskinesias, enhancing motor benefits, and delaying disease progression,″ “Combination serotonin specific reuptake inhibitor and serotonin 1A receptor partial agonist for reducing l‐dopa‐induced dyskinesia,” “Modulation of chitinase protein expression,” “Method of engineering and isolating adeno‐associated virus”.

## Data Availability

Data sharing is not applicable to this article as no datasets were generated or analyzed during the current study.
